# Rapid screening methods for yeast sub‐metabolome analysis with a high‐resolution ion mobility quadrupole time‐of‐flight mass spectrometer

**DOI:** 10.1002/rcm.8420

**Published:** 2019-05-02

**Authors:** Teresa Mairinger, Ruwan Kurulugama, Tim J. Causon, George Stafford, John Fjeldsted, Stephan Hann

**Affiliations:** ^1^ Department of Chemistry University of Natural Resources and Life Sciences – BOKU Vienna Muthgasse 18 1190 Vienna Austria; ^2^ Agilent Technologies 5301 Stevens Creek Blvd Santa Clara CA 95051 USA

## Abstract

**Rationale:**

The wide chemical diversity and complex matrices inherent to metabolomics still pose a challenge to current analytical approaches for metabolite screening. Although dedicated front‐end separation techniques combined with high‐resolution mass spectrometry set the benchmark from an analytical point of view, the increasing number of samples and sample complexity demand for a compromise in terms of selectivity, sensitivity and high‐throughput analyses.

**Methods:**

Prior to low‐field drift tube ion mobility (IM) separation and quadrupole time‐of‐flight mass spectrometry (QTOFMS) detection, rapid ultrahigh‐performance liquid chromatography separation was used for analysis of different concentration levels of dansylated metabolites present in a yeast cell extract. For identity confirmation of metabolites at the MS2 level, an alternating frame approach was chosen and two different strategies were tested: a data‐independent all‐ions acquisition and a quadrupole broad band isolation (Q‐BBI) directed by IM drift separation.

**Results:**

For Q‐BBI analysis, the broad mass range isolation was successfully optimized in accordance with the distinctive drift time to *m*/*z* correlation of the dansyl derivatives. To guarantee comprehensive sampling, a broad mass isolation window of 70 Da was employed. Fragmentation was performed via collision‐induced dissociation, applying a collision energy ramp optimized for the dansyl derivatives. Both approaches were studied in terms of linear dynamic range and repeatability employing ethanolic extracts of *Pichia pastoris* spiked with 1 μM metabolite mixture. Example data obtained for histidine and glycine showed that drift time precision (<0.01 to 0.3% RSD, *n* = 5) compared very well with the data reported in an earlier IM‐TOFMS‐based study.

**Conclusions:**

Chimeric mass spectra, inherent to data‐independent analysis approaches, are reduced when using a drift time directed Q‐BBI approach. Additionally, an improved linear dynamic working range was observed, representing, together with a rapid front‐end separation, a powerful approach for metabolite screening.

## 1 INTRODUCTION

Studies involving metabolomics rely strongly on the use of mass spectrometry technology in order to both confirm metabolite identity and quantify the small molecules in complex matrices ideally with minimal user intervention. To this end, instrumental and software advances within the last decade have made high‐resolution mass spectrometry (HRMS) an essential tool for supporting various metabolomics strategies including metabolite discovery and differential analysis. Nevertheless, obtaining a high‐resolution mass spectrum for an unknown metabolite is known not to be sufficient to enable confirmation of metabolite identity since, even if the chemical sum formula determination according to accurate mass and isotope pattern matching is accurate, the number of potential structure matches remains too high.^1^, ^2^, ^3^

Because of these major challenges faced for non‐targeted metabolome assessment, employment of additional analytical selectivity via the use of high‐resolution fragment (HR MS/MS) spectra can be used to support metabolite identification as a complementary and chemically informative descriptor.^4^, ^5^ In the case of HR MS/MS, data‐dependent acquisition strategies rely on isolation and fragmentation of precursor ions which have exceeded a user‐defined intensity threshold or any other measurable criteria such as isotopologue pattern, mass defect or the presence of a diagnostic ion.^6^ However, some limitations for non‐targeted assessment are also apparent. For example, some important precursor ions may not be selected by acquisition software for fragmentation particularly for closely eluting species with large differences in abundance, while difficulties in relative quantification (differential analysis) are often encountered. As an alternative, data‐independent algorithms (DIAs) allow multi‐event acquisitions to be carried out with fragmentation of all precursors regardless of abundance or spectral characteristics typically performed.^7^ In the crudest implementation alternating between low‐energy (0 V) and high‐energy collision cell events, this acquisition workflow generates a complex HR MS/MS dataset making data mining and correct association of fragments with precursor ions extremely challenging. In fact, DIA strategies are only made feasible due to innovative developments such as shifting of the *m*/*z* isolation window of the quadrupole to effectively constrain the range of precursors undergoing fragmentation at a given time point. Most successfully applied in the field of proteomics, strategies such as SWATH (sequential window acquisition of all theoretical spectra)^8^ and MS^E^ 9 allow rapid collection of HR MS/MS spectra corresponding to one of the constrained windows of precursor ions. Importantly, these windows can be defined by the user according to both retention time and *m*/*z* considerations, which has proven extremely effective for improving proteome coverage despite challenges arising from chimeric MS/MS spectra.

With the same goal of providing a complementary and chemically informative descriptor for metabolites, the combination of gas phase ion mobility separation with HRMS (i.e. IM‐HRMS) is now of emerging interest for non‐targeted metabolomics studies.^10^, ^11^, ^12^, ^13^ Of particular importance in this area is the use of mobility‐derived collision cross section (CCS) to support metabolite annotation, and also the possibility of harnessing the IM separation to support HR MS/MS workflows. Nesting of ion mobility separation prior to isolation and fragmentation (commercially available as IM‐QTOFMS instrumentation) may therefore provide a path for development of advanced DIA strategies that use a combination of mobility separation and variable quadrupole isolation to provide higher quality MS/MS spectra. In the case of metabolomics, the potential of this marriage of analytical approaches is well recognized, particularly as high‐quality CCS libraries are emerging,^14^ but can also be practically limited by the high structural diversity, low molecular masses and CCS of many metabolites. Therefore, rather than aiming for a “full picture” metabolomics workflow, strategies involving targeting of a large subset of the metabolome such as the examples elaborated in chemical derivatization workflows developed by the Li group^15^, ^16^, ^17^, ^18^ might provide a better means to exploit the selectivity of novel IM‐supported DIA approaches for metabolomics. In particular, differential ^12^C‐/^13^C‐isotope dansylation labeling of the metabolomic subset containing primary or secondary amines, or phenolic hydroxyl groups, has revealed more than 600 metabolites in human urine samples.

**Figure 1:**
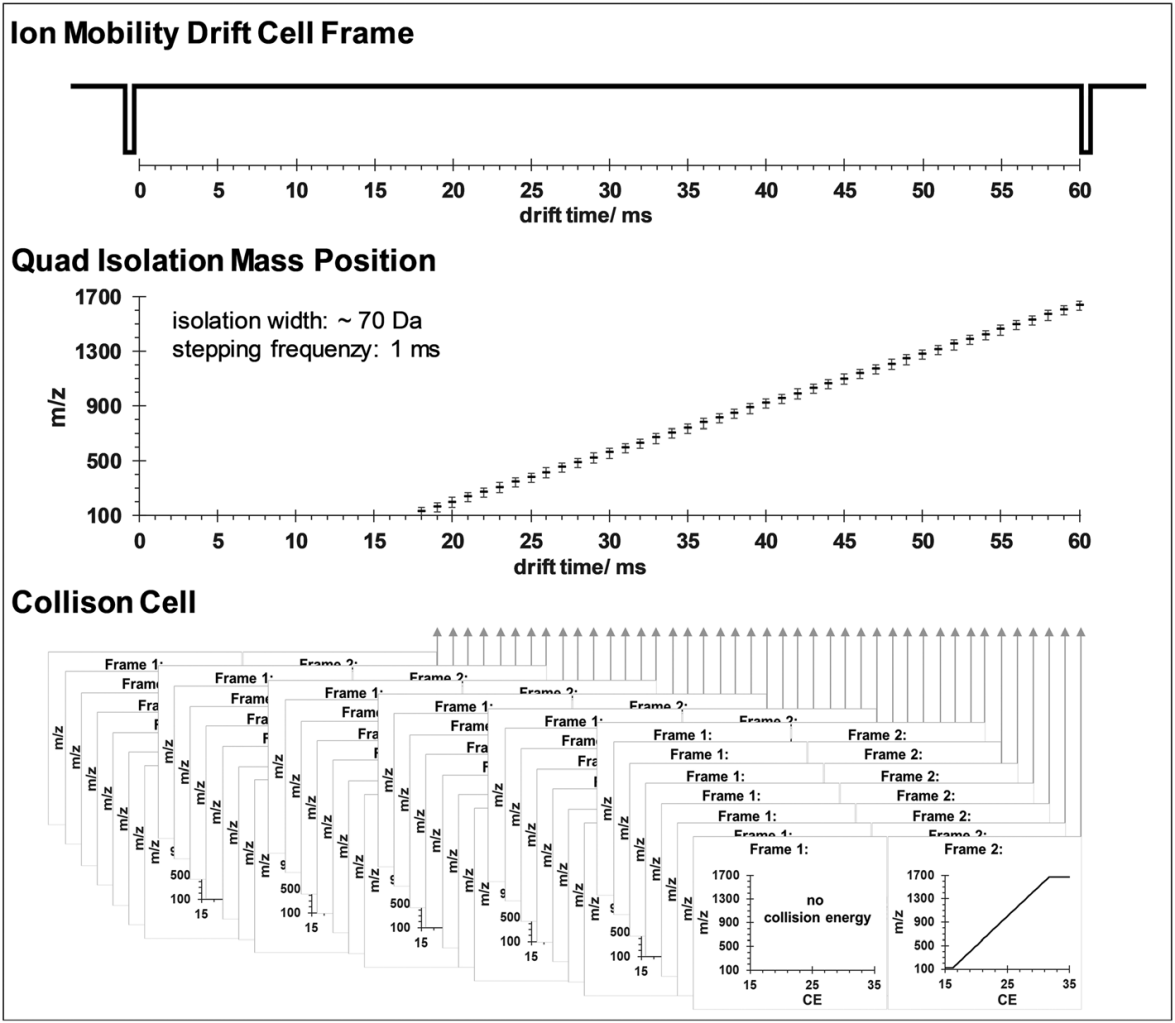
Schema of data‐independent ion mobility directed quadrupole broad band isolation (DIA IM‐Q‐BBI) approach. Within one IM frame, the quadrupole isolation can be ramped in a near‐continuous fashion to correspond with the drift times of precursor ions of interest. Precursors isolated by the combination of IM‐Q‐BBI are then subject to collision energy, allowing collection of cleaner fragment spectra using TOFMS

In this paper, we demonstrate that using advanced IM‐QTOFMS instrumentation equipped with a prototype continuous band quadrupole driver, the transmission settings and/or collision energy applied to dansylated precursors can be successfully directed by IM separation (i.e. the quadrupole transmission can be programmed to suit the drift times of dansylated precursors; see Figure 1 for more information). In this way, transmission and fragmentation of dansylated metabolites can be driven strongly toward only signals arising from precursors of interest based on their more predictable conformational ordering in the *m*/*z* versus drift time space. Moreover, employment of IM‐driven quadrupole isolation was expected to allow the speed of the liquid chromatography (LC) separation of dansylated metabolites to be increased substantially. It is worth noting that initial work on this type of instrumental setup was performed in the field of proteomics using a similar prototype of an Agilent 6560 IM‐QTOFMS.^19^ A similar concept was also realized using other vendor instrumentation, namely Waters Vion IM QTofMS and its SONAR™ technology, and successfully tested in the field of drug metabolism and pharmacokinetics.^20^, ^21^

The present work provides a detailed performance comparison of a fast LC separation (<5 min cycle time) in combination with an IM‐QTOFMS platform using two IM‐DIA modes for the analysis of dansylated metabolites present in yeast cell extracts.

## 2 EXPERIMENTAL

### 2.1 Chemicals

Acetonitrile, methanol, water, acetone, all of LC‐MS grade (≥99.0% purity), dansyl chloride (≥99.0% purity), Supelclean™ PSA SPE bulk packaging, sodium bicarbonate (≥99.7% purity) and sodium carbonate (≥99.5% purity) were purchased from Sigma Aldrich (St Louis, MO). Formic acid 98–100% Suprapur was purchased from Merck (Merck Millipore, Darmstadt, Germany). All metabolite standards (glycine, alanine, serine, ortho‐acetylserine, proline, valine, homoserine, threonine, isoleucine, leucine, asparagine, aspartate, glutamine, glutamate, lysine, methionine, histidine, arginine, cystine, homocysteine, *S*‐adenosylhomocysteine, cysteine, l‐cystathionine, cysteinylglycine, glutamylcysteine, phenylalanine, tyrosine, tryptophan, glutathione‐oxidized, glutathione‐reduced, cis‐aconitate, citrate, isocitrate, alpha‐ketoglutarate, oxaloacetic acid, malate, succinate, fumarate, dihydroxyisovalerate, ketoisovalerate, pyruvate, lactate, fructose, galactose, mannose, glucose, mannitol, gluconate, inositol, erythritol, trehalose, ribose, xylose, 2‐phosphoglycerate, 3‐phosphoglycerate, phosphoenolpyruvate, dihydroxyacetonephosphate, erythrose‐4‐phosphate, ribose‐5‐phosphate, ribulose‐5‐phosphate, fructose‐1‐phosphate, fructose‐6‐phosphate, glucose‐1‐phosphate, glucose‐6‐phosphate, mannose‐6‐phosphate, 6‐phosphogluconate, fructose‐1,6‐bisphosphate, sedoheptulose‐7‐phosphate, mannitol 1‐phosphate, adenine, uridine, thymine, uracil, inosine, cytosine, guanine, guanosine, 5’CMP, 5’UMP, 5’AMP, 3’AMP, 5’IMP, 5’GMP, ADP, GDP, riboflavin) were purchased from Sigma Aldrich and Merck. Single standard solutions were prepared in LC‐MS grade water containing suitable additives (0.1 M HCl or 0.1 M NaOH) when necessary. Ethanolic cell extracts of *Pichia pastoris* were purchased from ISOtopic solutions (Vienna, Austria). ESI low‐concentration tune mix for tuning, mass calibration and single‐field CCS calibration was obtained from Agilent Technologies (Santa Clara, CA, USA).

### 2.2 Sample preparation and derivatization

An equimolar mixture of the aforementioned metabolites was prepared from a single standard solution and evaporated under reduced pressure using a GeneVac EZ2 solvent evaporation system to a final amount of 10 nmol. Prepared metabolite mixtures were stored at −80°C and reconstituted and diluted on the day of analysis to the following concentration levels: 0.1, 0.5, 1, 5, 10, 25, 50 and 100 μM.

To account for matrix effects, an ethanolic yeast cell extract (cell dry weight = 15 mg) was employed. After a dilution step of 1:50, 100 μL aliquots were evaporated and reconstituted in 100 μL of the respective metabolite standard solutions containing a final cell dry mass of 3.0 μg. Additionally, one aliquot was reconstituted with LC‐MS grade water.

For analysis of amine‐carrying metabolites, dansylation was chosen as the derivatization method. As a starting point for the derivatization procedure, the conditions of Li and coworkers^15^ were adapted. The derivatization was conducted as follows: 100 μL sample volume was mixed with 100 μL of 0.5 M Na_2_CO_3_/NaHCO_3_ buffer (pH 9.50) in order to maintain an alkaline pH of 9.5. After vortexing, 100 μL of dansylation reagent (20 mg mL^−1^ dansyl chloride in 90:10 acetonitrile:acetone solution) was added and vortexed again. A 2 mL amber glass vial was used as reaction vessel and in the following the mixture was incubated for 1 h at 60°C in an agitator (250 rpm). The derivatization reagent was prepared freshly, whereas the buffer solution was kept at 4°C and pH was always controlled before usage. To remove the excess of non‐reacted derivatization reagent, an aliquot of 250 μL was transferred into another 2 mL amber glass vial, where approximately 25–30 mg of PSA (primary secondary amine) SPE bulk material was weighed in beforehand, and was incubated for another 15 min at 60°C in an agitator (250 rpm). Finally, aliquots of 200 μL were carefully transferred into HPLC vials with inserts and were stored for a maximum of 24 h at 6°C in a cooled tray until analysis.

### 2.3 LC/ESI‐IM‐QTOFMS analysis

For sample injection and chromatographic separation a Gerstel Dual Rail multipurpose sampler (MPSII, Gerstel, Germany) and an Agilent 1290 Infinity II HPLC system was used. IM mass spectrometry analyses were carried out using an Agilent 6560 IM‐QTOF instrument equipped with a uniform low‐field drift tube IM device and a high‐resolution mass analyzer. An Agilent dual jet stream source was used as ESI source. This instrument was modified with a prototype broad band isolation quadrupole driver, enabling a drift time directed mass isolation.

For separation of dansylated metabolites, an Acquity UPLC BEH C18 column (1.7 μm, 1.0 × 50 mm) was employed using water containing 0.1% formic acid (eluent A) and methanol containing 0.1% formic acid (eluent B) for elution. The separation was operated at a column temperature of 40°C applying the following gradient elution: starting conditions were adapted to the solvent composition of the derivatized sample, hence the gradient elution started with 30% organic phase and was held constant for 0.5 min, then increasing to 100% B within 2.5 min. To flush the column, 100% B was held until 3.9 min, returning at 4 min to the starting conditions of 30% B and equilibrating the column for 1 min. A flow rate of 300 μL min^−1^ was employed and 1 μL sample volume was injected partial loop (total loop volume 5 μL). An extracted ion chromatogram of a 1 μM multi‐metabolite mixture in ethanolic yeast extract subjected to dansylation can be found in Figure S1 (supporting information).

For MS detection, mass spectra between 50 and 1700 *m*/*z* were recorded in positive polarity mode using the 2 GHz extended dynamic range mode. For ionization the following settings were used: drying gas temperature, 225°C; drying gas flow, 13 L min^−1^; nebulizer pressure, 40 psig; sheath gas temperature, 350°C; sheath gas flow, 12 L min^−1^; capillary voltage, 4000 V; nozzle voltage, 500 V; fragmentor voltage, 400 V; octapole 1 RF, 750 V.

For identity confirmation of metabolites at the MS2 level, two different analytical strategies relying on a low‐field drift tube IM‐QTOFMS were tested.

Here, either a data‐independent ion mobility all‐ions (DIA IM‐AI) acquisition or a non‐commercially available prototype of quadrupole broad band isolation (Agilent Technologies) directed by IM‐drift separation (DIA IM‐Q‐BBI) was employed. In the case of DIA IM‐AI, the alternating IM frame mode is switching between MS1 and MS2 and employs an optimized CE ramp within each IM transient. In the other case, namely DIA IM‐Q‐BBI, also an alternating IM frame mode is applied. However, here, the correlation between drift time and *m*/*z* 22 is made use of to direct the quadrupole mass isolation according to the drift time. Thus, for each ion mobility frame, lasting 60 ms, the quadrupole, which is situated after the drift tube, is set according to the *m*/*z* value to be expected at the respective drift time in steps of 1 ms. Hence this broad band quadrupole isolation ramp was also optimized according to the compound class. The second frame employs again an optimized CE ramp. The approach is schematically illustrated in Figure 1.

The mass spectrometer was mass‐calibrated before every measurement using the Agilent ESI Tune solution. This tune mix was also employed for single field CCS calibration as described previously.^23^ IM RealTime viewer, a standalone utility software (Agilent Technologies), was employed for setting up the quadrupole mass isolation ramp with respect to the drift time distribution.

Data evaluation for optimization purposes was performed using MassHunter Qualitative Analysis (B08.00) and MassHunter IM‐Browser (B08.00). For molecular feature finding, Agilent MassProfinder (B08.00) was employed. For quantitative evaluation, Skyline (4.1.0.11796) was used.

For IM separation prior to MS detection the following parameters were used: 1574 V at the drift tube entrance, 224 V at the drift tube exit, 217.5 V at rear funnel entrance, 45 V at rear funnel exit, 60 ms total drift time, 10 ms trapping time, 300 μs trap release time, 16 IM transients per frame, leading to a cycle time of 0.96 s. For Q‐BBI, a shifting isolation width of approximately 70 Da was employed. However, the prototype quad driver showed slight decrease in isolation width with increasing *m*/*z* (78 at *m*/*z* 322, 71 at *m*/*z* 622 and 69 at *m*/*z* 922). A survey experiment was utilized to ascertain the number of mass isolation windows and the maximum mass range for the mass isolation ramp. The BBI quadrupole ramp method was set to have a 1 ms dwell time, that is, each mass isolation window is active for a 1 ms period before the mass window is shifted to the next isolation window. The total number of isolation windows employed depends on the sample and the width of mass isolation window. Optimized conditions for drift time directed Q‐BBI and collision energy ramp for dansylated compounds are presented in Table 1.

**Table 1:** Optimized conditions for drift time directed Q‐BBI and respective collision energy for dansylated metabolites using low‐field drift tube IM‐QTOFMS

Drift time (ms)	m/z	Collision energy (eV)
*18*	*130*	*16*
22	270	18
25	410	19
29	560	21
33	710	22
37	850	24
41	990	25
45	1130	26
49	1280	28
53	1420	29

## 3 RESULTS

### 3.1 Optimization of collision energy ramps

To optimize the collision energy ramps, the equation typically used (CE = (slope × precursor *m*/*z*)/100 + offset) was varied in its values for slope and offset (1, 3, 5 and 5, 10, 15, 20, respectively). If the precursor ion was present at approximately 10% of the base peak, a condition was considered as optimal. Decisions on optimal parameters were made via visual inspection of MS2 spectra of eight dansylated compounds (1DNS‐glycine, 1DNS‐serine, 1DNS‐uracil, 1DNS‐proline, 1DNS‐aspartate, 1DNS‐adenine, 1DNS‐phenylalanine and 2DNS‐glutamine) using MassHunter Qualitative Analysis (B08.00, Agilent Technologies). An offset of 15 and a slope value of 1 yielded overall acceptable fragmentation patterns and these settings were thus chosen for all acquisition modes to calculate the collision energy ramp.

### 3.2 Optimization of drift time directed Q‐BBI

As pointed out previously, DT‐IM and MS are not considered as orthogonal techniques, though they will deliver complementary information, since there is a clear correlation between mass‐to‐charge ratio and drift times of particular molecular classes in low‐field drift time IM instrumentation. When dealing with research areas where analytes are characterized by similar building blocks, e.g. lipidomics, the relationship between mobility and mass‐to‐charge ratio can be exploited for compound class prediction.^22^ However, in metabolomics, this is hardly the case due to the greater chemical diversity and lack of well‐defined drift time to *m*/*z* correlations. In order to increase selectivity and thereby tackle the challenge of complex biological matrices, derivatization proved to be an effective approach. Dansylation is already well established, also in metabolomics,^15^, ^16^, ^17^, ^18^ and apart from its benefits in terms of improved chromatographic separation on reversed‐phase columns and improved electrospray response, the additional aromatic ring structure leads to a more pronounced correlation between drift time and *m*/*z*, compared to non‐derivatized amine‐carrying metabolites.

To guarantee effective and selective sampling of dansylated analytes in terms of *m*/*z*, the Q‐BBI needs to be optimized according to the drift time versus *m*/*z* trendline. For assessing this correlation and determine which masses can be expected at which drift times, dansylated solvent standard samples (approximately 8 and 16 pmol on column) were measured in DIA‐IM‐AI mode and the molecular feature finding algorithm using MassProfinder was employed. Molecular features, passing the filters (mass filter > *m*/*z* 250, drift time filter >15 ms, minimum number of ions 2, quality score > 70), were plotted according to *m*/*z* versus drift time and a linear regression was established. The regression coefficient obtained for the linear regressions of these filtered molecular features was 0.95 ± 0.02. This was considered as promising for subsequent experiments and validates thereby our working hypothesis.

Since the measured drift time represents the total time an ion needs from the beginning of the drift tube until it finally arrives at the TOF pusher region and does not reflect the actual arrival time of the ion entering the quadrupole, a correction must be applied. This correction factor is empirically determined as a fraction of the total drift time.

In the next step, in order to describe the drift time versus *m*/*z* trendline for dansylated compounds, in total nine points were evenly distributed over the drift time range and the respective *m*/*z* values were calculated according to the linear regression equation and rounded. The drift times were then corrected by using 92, 95 or 98% of the drift time. Subsequently, these drift time versus *m*/*z* trendlines were tested for Q‐BBI directed by IM separation for dansylated compounds.

To eliminate differences in outcomes due to the derivatization procedure, samples were taken from the very same vials for each tested condition. Stability of derivatives was tested beforehand and is consistent with literature findings.^15^ Evaluation of the optimal drift time correction was performed according to the maximum number of molecular features detected (66, 49 and 24 molecular features were detected for 98, 95 and 92%, respectively) and a value of 98% of the drift time was chosen as DT correction factor for the drift time versus *m*/*z* trendline and was also applied to the collision energy ramp.

The mass over charge versus drift time plot of the detected molecular features employed for optimization can be found in Figure S2 (supporting information), whereas the optimized drift time versus *m*/*z* trendline and collision cell ramp is presented in Table 1.

### 3.3 Evaluation of DIA IM‐AI and DIA IM‐Q‐BBI

**Figure 2:**
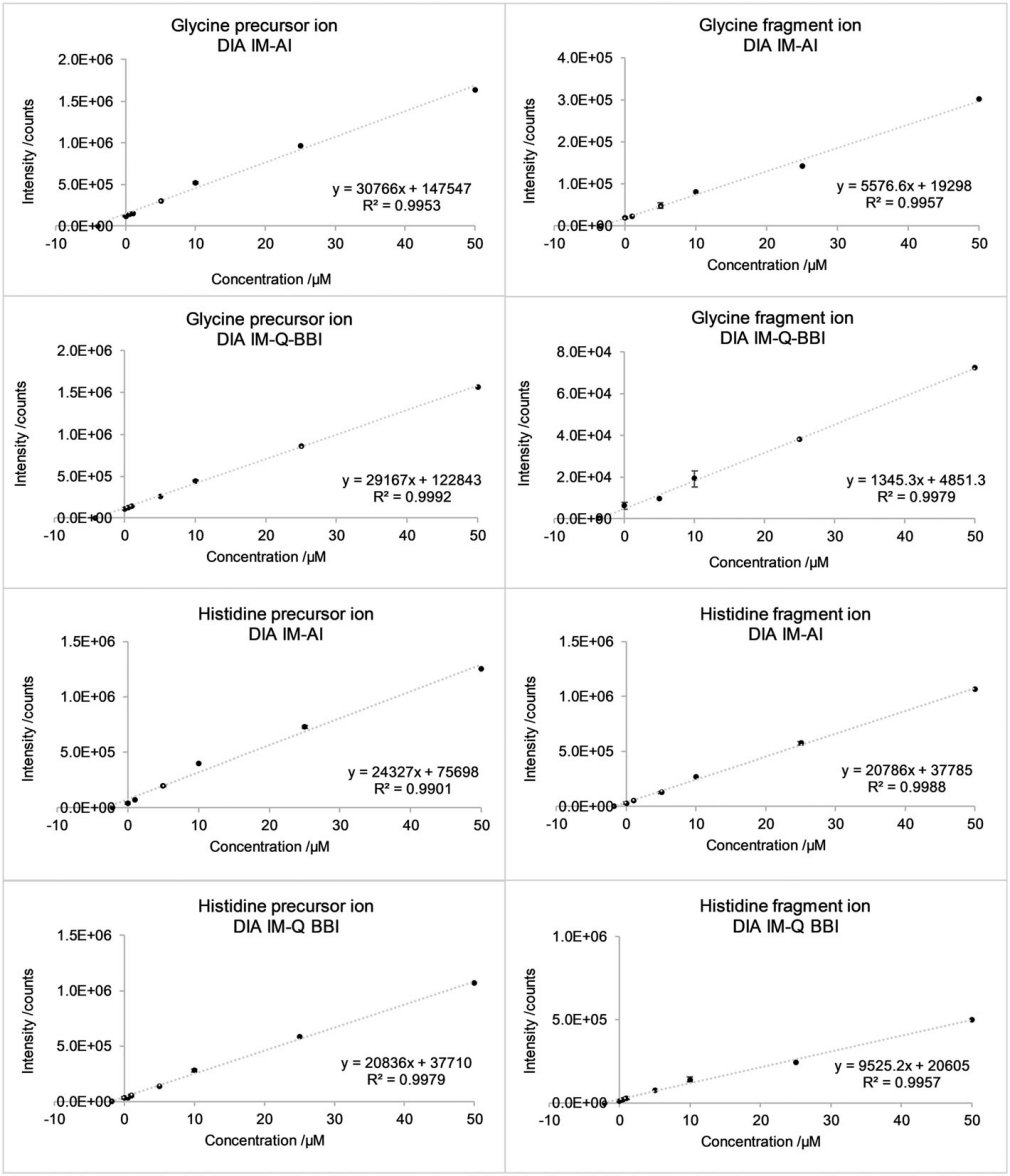
Calibration curve of two model compounds, namely the amino acids glycine and histidine, for the precursor ion, [M + H]^+^, as well as a selective fragment ion, measured either via the IM‐AI approach or using IM‐Q BBI. The values shown on the x‐axis reflect the concentration of the solution employed for reconstituting the dried yeast cell extract in the sample vial

To evaluate fitness for purpose of the two IM‐based approaches in terms of linear dynamic range as well as figures of merit regarding repeatability of drift time, accurate mass, peak area and ratio of precursor ion to fragment ion, two representative compounds were selected and extracted with a targeted approach, namely glycine and histidine. Figure 2 shows the calibration curves obtained for the precursor and fragment ions of the two compounds, applying either the IM‐AI approach or the IM‐Q‐BBI approach. The fragment ion was inferred by employing MassFrontier, version 7.0, an in‐silico fragmentation software. Fragment ions were then checked for consistency according to their isotope pattern as well as accurate mass. For glycine, C_13_H_14_N_2_O_4_S, *m*/*z* 294.0669 was employed, whereas for histidine, C_5_H_9_N_3_, *m*/*z* 110.0712 was used. The concentration range depicted refers to the concentration of the analytical standard solution that was employed for reconstituting a defined amount of yeast cell extract (3.0 μg) and ranges from 0.1 μM up to 50 μM. It can be seen that the correlation coefficient is generally in the same range for both approaches. Besides, higher calibration points of the precursors in the IM‐AI approach indicate quadratic response, suggesting that using IM‐Q‐BBI shows benefits in terms of working range, achieving easily three orders of magnitude.

Table 2 presents the figures of merit of the two compounds. Here, five repeat injections of a 1 μM analytical standard solution reconstituting 3.0 μg cell dry weight of yeast cell extract were performed. Excellent repeatability was observed in the case of drift times, being in good agreement with values reported from Stow et al.^23^ Regarding the peak area, a good repeatability for both precursor and fragment ions was achieved. However, it is worth mentioning that for glycine a clear difference in peak area repeatability between precursor and fragment ions was observed. This can be explained by the rather low peak area of the fragment ion and hence ion counting statistics.

**Table 2:** Analytical figures of merit of drift time‐directed quadrupole isolation obtained with a prototype broad band driver. Precision was calculated for five independent sample injections from ethanolic extracts of Pichia pastoris spiked with 1 μM metabolite mixture

	Glycine precursor ion				Glycine fragment ion					Histidine precursor ion				Histidine fragment ion				
	No. of IM transients	DT	m/z	Peak area	No. of IM transients	DT	m/z	Peak area	Ratio fragment/precursor %	No. of IM transients	DT	m/z	Peak area	No. of IM transients	DT	m/z	Peak area	Ratio fragment/precursor %
Sample		ms		Counts × min		ms		Counts × min			ms		Counts × min		ms		Counts × min	
1 μM in P.pastoris_a	272	22.75	309.0898	145101	272	21.54	294.0664	6165	4.25	306	25.57	389.1267	56349	306	24.55	110.0715	24529	44
1 μM in P.pastoris_b	272	22.75	309.0894	143712	272	21.54	294.0670	6314	4.39	306	25.62	389.1264	51459	306	24.55	110.0712	29351	57
1 μM in P.pastoris_c	272	22.75	309.0899	140700	272	21.54	294.0652	6809	4.84	306	25.71	389.1266	55009	306	24.55	110.0710	25430	46
1 μM in P.pastoris_d	272	22.77	309.0900	142140	272	21.55	294.0666	5217	3.67	306	25.52	389.1265	53406	306	24.55	110.0711	27826	52
1 μM in P.pastoris_e	272	22.75	309.0896	137265	272	21.54	294.0662	4686	3.41	306	25.58	389.1275	47066	306	24.55	110.0712	25722	55
**Average**		**22.75**	**309.0897**	**141784**		**21.54**	**294.0663**	**5838**	**4.11**		**25.60**	**389.1267**	**52658**		**24.55**	**110.0712**	**26572**	**50.8**
**Standard deviation**		**0.01**	**0.0002**	**3018**		**0.004**	**0.0007**	**864**	**0.57**		**0.071**	**0.0004**	**3620**		**0**	**0.0002**	**1968**	**5.6**
**RSD (%)**		**0.04**		**2.1**		**0.02**		**15**	**14**		**0.3**		**7**		**0.0**		**7**	**11**

## 4 DISCUSSION

The presented approach aims for a significant enhancement of analytical selectivity in non‐targeted analysis by combining dansylation of metabolites and rapid UHPLC with prototype Q‐BBI directed by drift‐tube IM mass spectrometry. Apart from the selectivity enhancement by derivatization, one of our working hypotheses relies on the chimeric spectra obtained when using an AI approach, whereas, when employing a BBI using this prototype quadrupole, the analytes of interest can be selected before the collision cell leading to less overlap with matrix constituents. As a consequence, this also leads to cleaner fragmentation spectra. This can be clearly observed in the fragment spectra obtained by the two approaches (see Figure S3, supporting information). A minor advantage can be also seen in data storage, since data files using the IM‐Q‐BBI approach are roughly 40% smaller compared to the IM‐AI approach In terms of sensitivity, the precursor ion counts achieved with the two approaches were similar; however, the IM‐AI mode revealed a factor of 2–4 higher sensitivity for the fragment ions. Transmission of fragment ions will be improved in future work by optimizing the *m*/*z* versus drift time ramp for quadrupole isolation in a high‐energy step.

**Figure 3:**
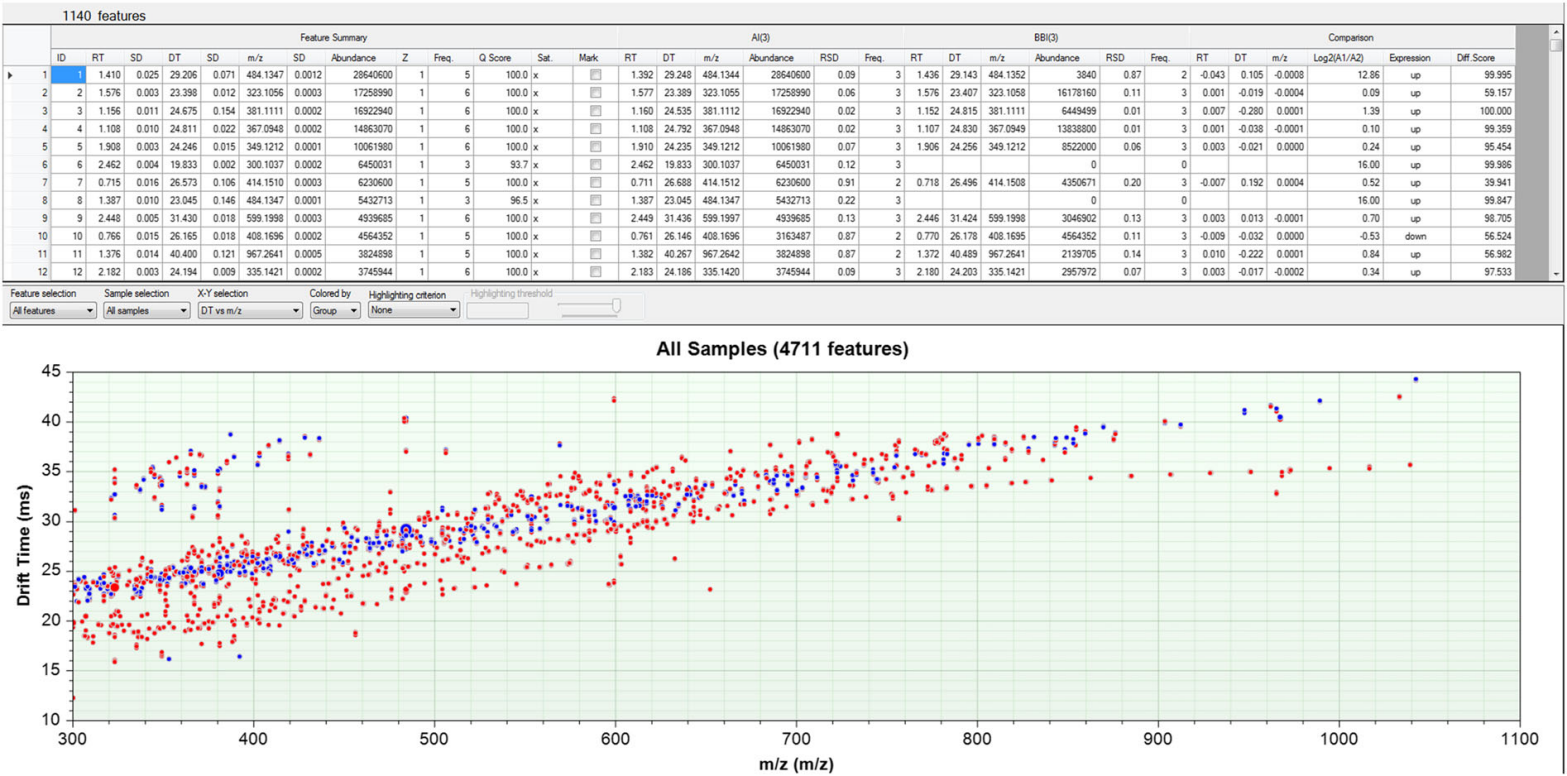
Molecular features detected by LC/IM‐QTOFMS in all‐ions mode (IM‐AI, red) versus drift time directed quadrupole isolation (IM‐Q‐BBI, blue) in ethanolic extracts of the yeast Pichia pastoris (n = 3)

In Figure 3 molecular features, sufficing the aforementioned quality criteria, detected by either the non‐targeted IM‐AI (in red) or the IM‐Q‐BBI (blue) approach are plotted according to their drift time‐to‐*m*/*z* correlation. The effective selectivity enhancement for the BBI can be clearly seen as the features are constrained to the conformational region (i.e. drift versus *m*/*z* trend) of interest, thus providing higher confidence in compound identity confirmation. Precursor ions not effectively isolated by the combined IM‐BBI approach are filtered out. Despite the more complex nature of the experiment, the fate of ions that are outside of the *m*/*z* (quadrupole) isolation window at any given time point is the same as for a QTOF‐only experiment.

**Figure 4:**
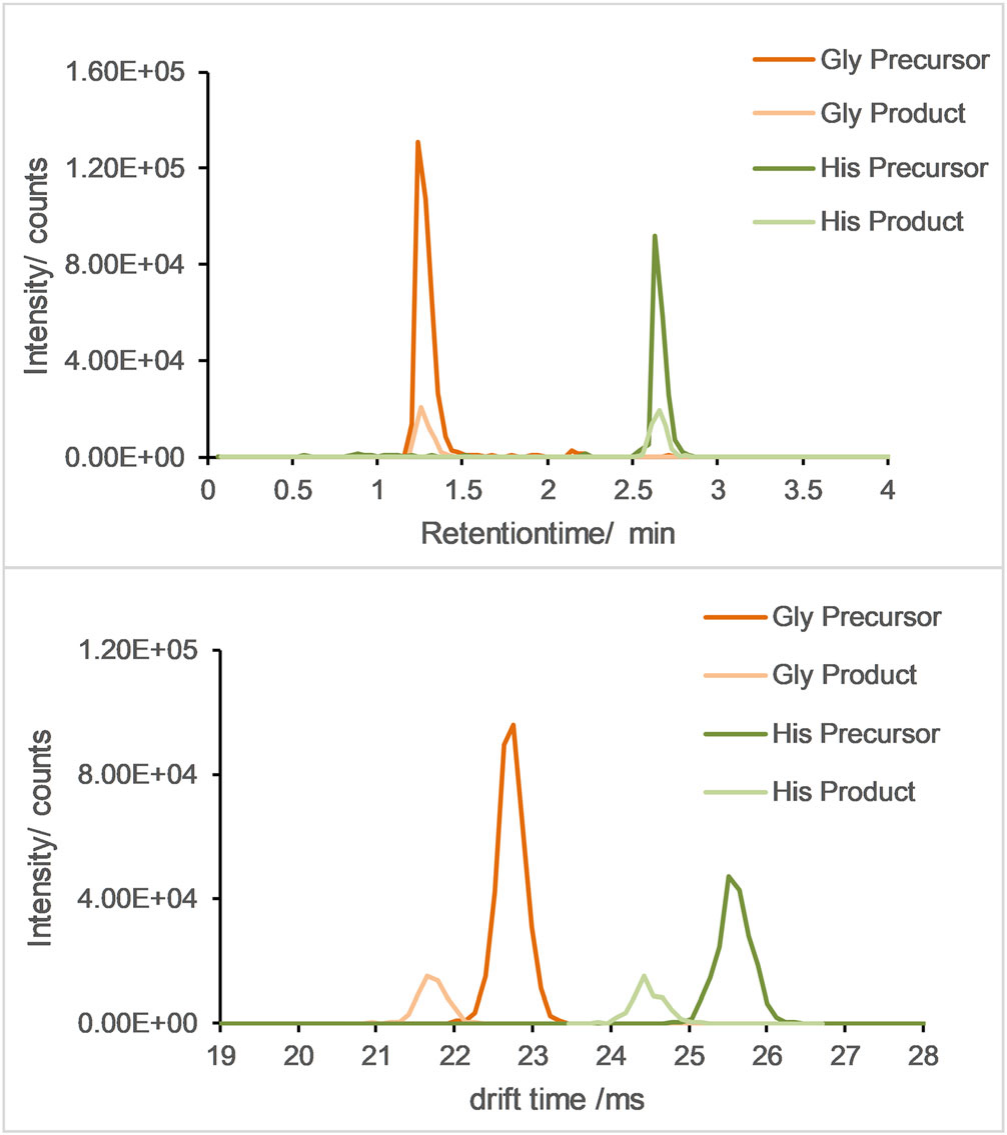
Accurate mass and retention time extracted ion current (upper panel) and drift spectra of derivatized glycine and histidine precursor and fragment ions (5 μM multi‐metabolite mixture in ethanolic yeast extract). The negative drift time shift of the product ions compared to the precursor ions can be explained by the higher acceleration of the lighter fragment ions caused by the field gradient over the collision cell as well as the ion beam compressor region during the high‐energy step. Since the measured drift time is a function of ion drift inside as well as outside the drift tube, t_0_ of lighter fragment ions is smaller, leading to a negative drift time shift. Precursor and product ions are automatically aligned during data evaluation

As evident from Table 2, the drift times between precursor ion and fragment ion have a negative shift of nearly 1 ms. This negative shift can be explained by the fact that under the accelerating electric field smaller ion fragments move faster through the collision cell and the ion beam compressor region during high‐energy steps than larger precursor ions; hence *t*_0_, i.e. the time ions spend traveling though the instrument, outside the drift tube, is different. It is noteworthy that this drift time shift is a function of the collision energy used and the mass of the fragment ion. Considering that *t*_0_ values are typically in the range 5–8 ms in the present instrument, a shift of approximately 4.5% is reasonable. This observation is also illustrated in Figure 4. In the upper panel of the figure, a perfect alignment of precursor and fragment ions in terms of retention time is shown, whereas in the lower panel the negative drift time shift of approximately 1 ms is plotted. It is worth mentioning that during feature finding in Mass Profiler software, the MS1 and MS2 frames are still automatically aligned, since a slightly wider drift selection window is used for precursor and fragment ion alignment.

## 5 CONCLUSIONS

Employment of a novel sub‐metabolome screening workflow based on dansylation chemistry followed by an advanced drift time directed BBI strategy allows the determination of small metabolites over several orders of magnitude within yeast extract samples. Using this new workflow, fragment/precursor ratios obtained via alternating frames acquisition with quadrupole and collision energy ramping were found to be stable over the working range studied. Evaluation of fragment intensities for quantification purposes also extends the analytical working range for this application and is therefore promising in the context of fold change analysis for a wider range of metabolomics approaches including future method developments and derivatization strategies for consideration of other metabolite classes. Moreover, in comparison to the IM‐AI mode, the more advanced IM‐Q‐BBI approach yielded improved linearity, a wider working range for the investigated precursor ions and fewer MS artefacts in final datasets.

## Supporting information

Rapid screening methods for yeast sub‐metabolome analysis on a high‐resolution IM‐QTOF mass spectrometer

**Figure S1**: UHPLC separation of dansylated compounds (1 μM multi‐metabolite mixture in ethanolic yeast extract)

**Figure S2**: Drift time versus m/z plot for dansylated compounds. Molecular features depicted here are representing the combined results of two dansylated solvent standards (8 and 16 pmol on column).

**Figure S3**: CID fragment spectra obtained by LC‐IM‐QTOFMS in a non‐targeted IM‐AI or the IM‐Q‐BBI approach applying the fragmentation conditions described in the experimental section. The spectra where extracted in the retention time interval of 1.15–1.45 min (retention time window of glycine) of an ethanolic extract of the yeast Pichia pastoris, which was spiked with 10 μM glycine.
