# Supplementary material for: Rapid screening methods for yeast sub‐metabolome analysis with a high‐resolution ion mobility quadrupole time‐of‐flight mass spectrometer
Source: Rapid Commun Mass Spectrom. 2019 May 2;33(Suppl Suppl 2):66–74. doi: 10.1002/rcm.8420 (PMC6618165; doi:10.1002/rcm.8420)
Supplement: Supplementary file 1 — Rapid screening methods for yeast sub‐metabolome analysis on a high‐resolution IM‐QTOF mass spectrometer [file RCM-33-66-s001.docx]

**Rapid screening methods for yeast sub-metabolome analysis on a high-resolution IM-QTOF mass spectrometer**

Teresa Mairinger^1, §^, Ruwan Kurulugama^2^, Tim J. Causon^1^, George Stafford^2^, John Fjeldsted^2^, Stephan Hann^1*^

^1^Department of Chemistry, University of Natural Resources and Life Sciences - BOKU Vienna, Muthgasse 18, 1190 Vienna, Austria

^§^current address: EAWAG, Swiss Federal Institute of Aquatic Science and Technology, Ueberlandstrasse 133, 8600 Dübendorf, Switzerland

^2^Agilent Technologies, 5301 Stevens Creek Blvd, Santa Clara, CA 95051, USA

*corresponding author: email : stephan.hann@boku.ac.at

# Supplementary information


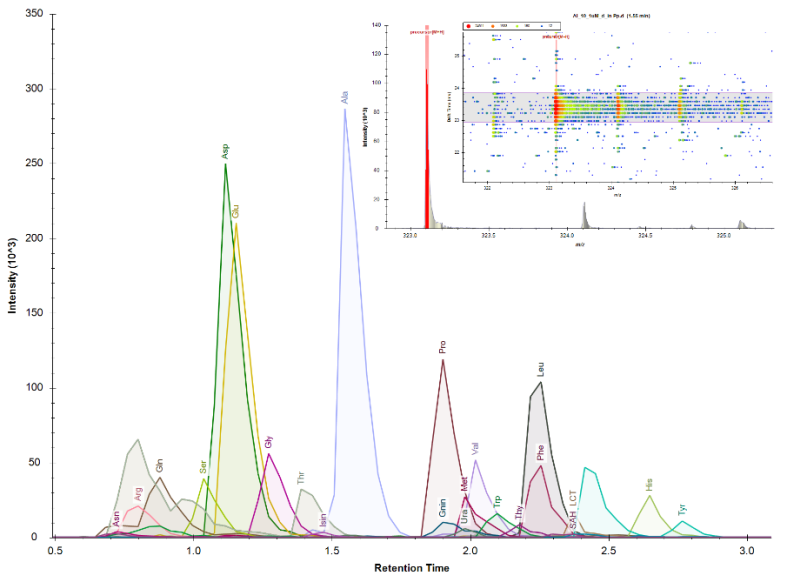


Figure S1: UHPLC separation of dansylated compounds (1 µM multi-metabolite mixture in ethanolic yeast extract)


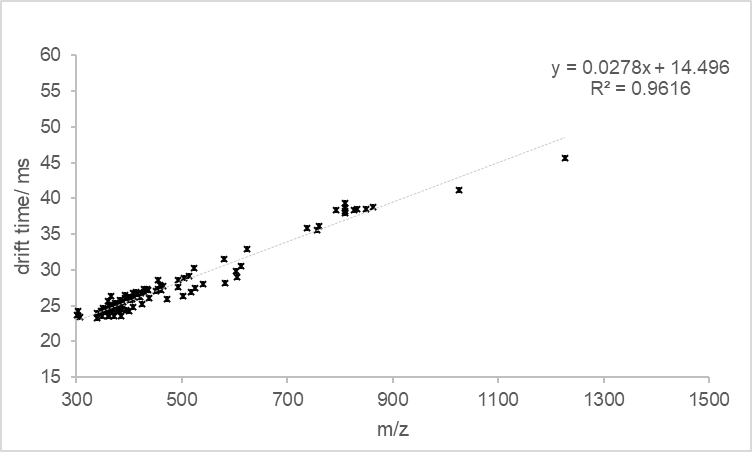


Figure S2: Drift time versus m/z plot for dansylated compounds. Molecular features depicted here are representing the combined results of two dansylated solvent standards (8 and 16 pmol on column).

Figure S3: CID fragment spectra obtained by LC-IM-QTOFMS in a non-targeted IM-AI or the IM-Q-BBI approach applying the fragmentation conditions described in the experimental section. The spectra where extracted in the retention time interval of 1.15 – 1.45 min (retention time window of glycine) of an ethanolic extract of the yeast *Pichia pastoris*, which was spiked with 10 µM glycine.
