# Supplementary figures and images for: Rapid screening methods for yeast sub‐metabolome analysis with a high‐resolution ion mobility quadrupole time‐of‐flight mass spectrometer
Source: Rapid Commun Mass Spectrom. 2019 May 2;33(Suppl Suppl 2):66–74. doi: 10.1002/rcm.8420 (PMC6618165; doi:10.1002/rcm.8420)

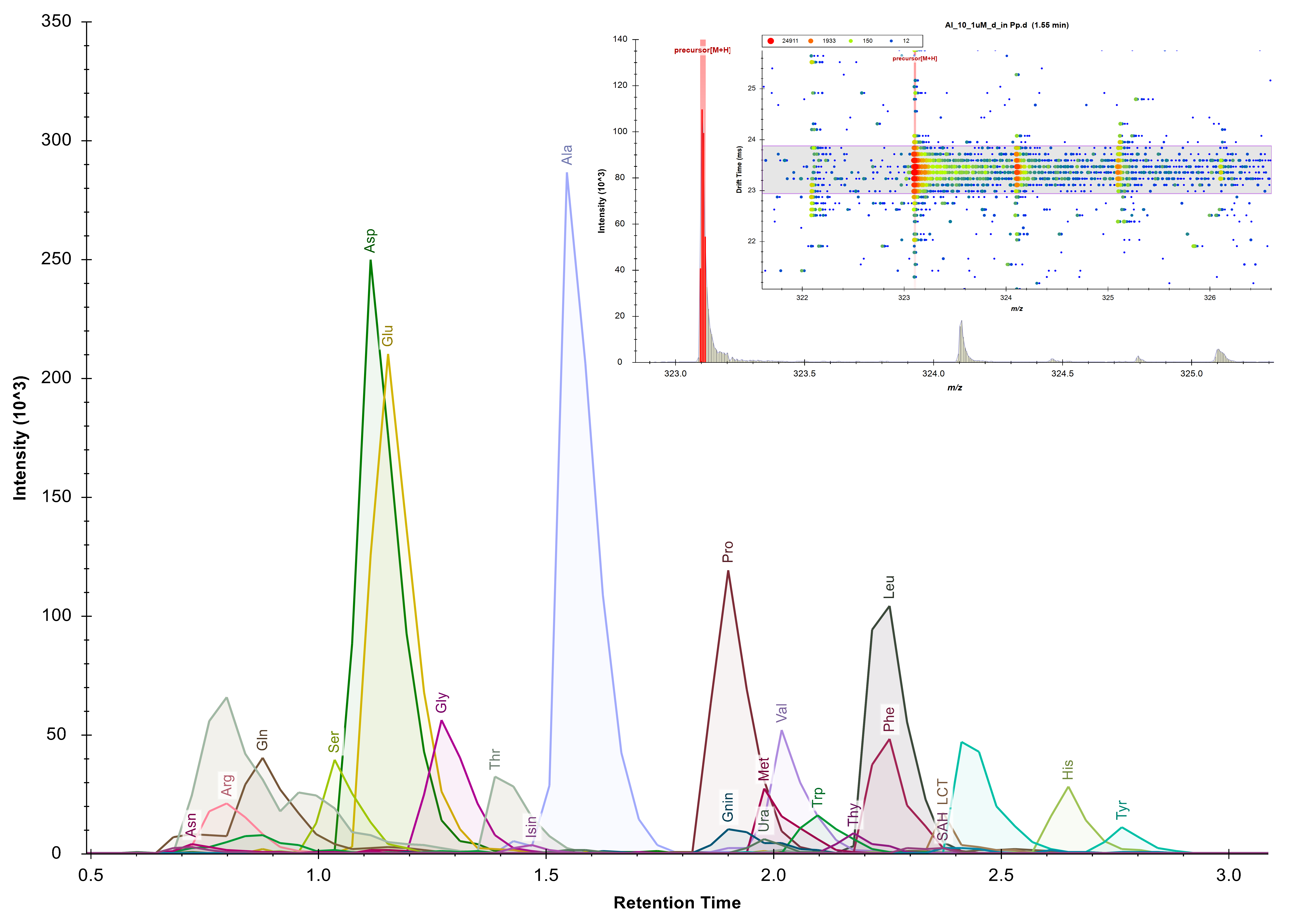

Supplement: Supplementary file 2 — Figure S1: UHPLC separation of dansylated compounds (1 μM multi‐metabolite mixture in ethanolic yeast extract) [file RCM-33-66-s002.tif]

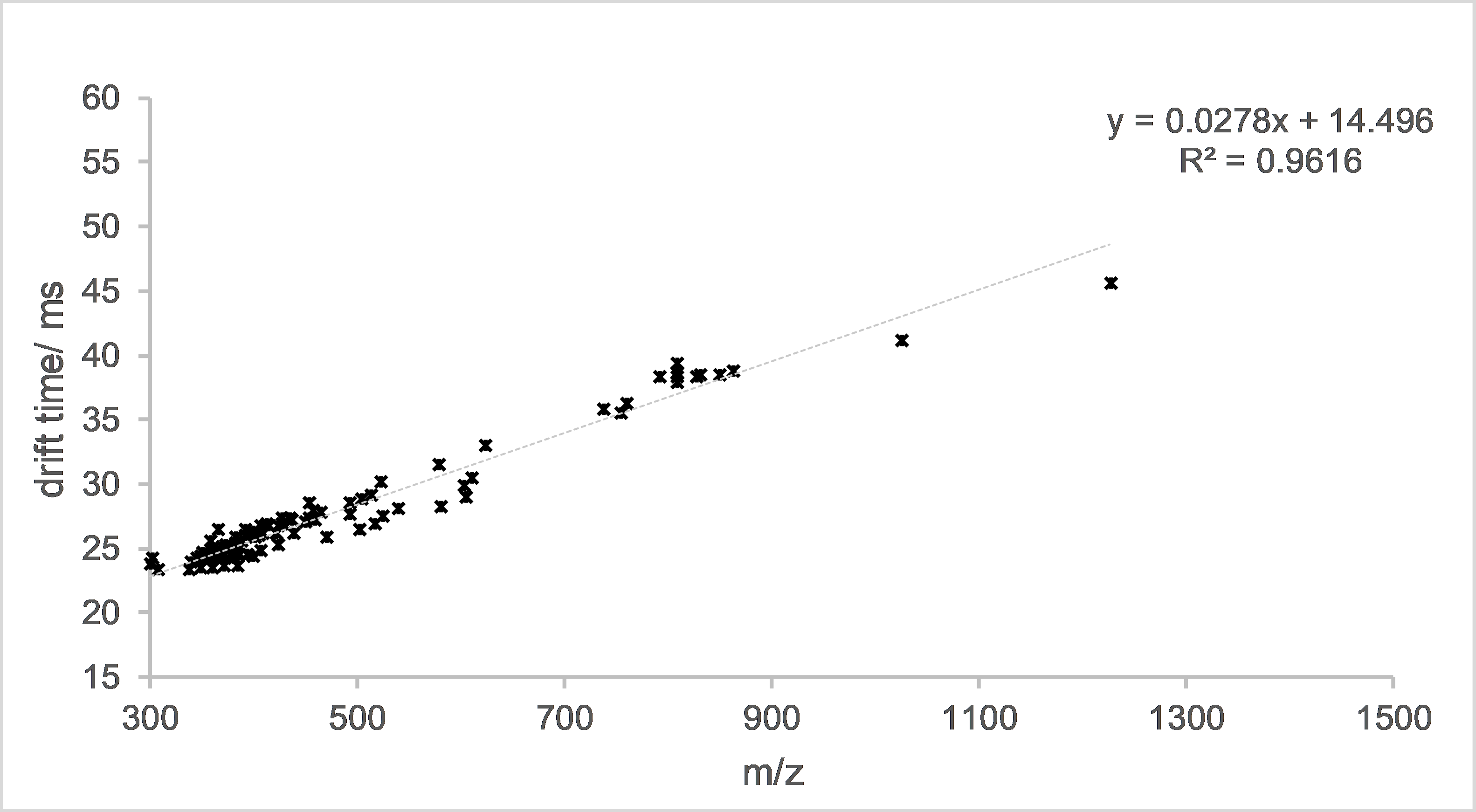

Supplement: Supplementary file 3 — Figure S2: Drift time versus m/z plot for dansylated compounds. Molecular features depicted here are representing the combined results of two dansylated solvent standards (8 and 16 pmol on column). [file RCM-33-66-s003.tif]

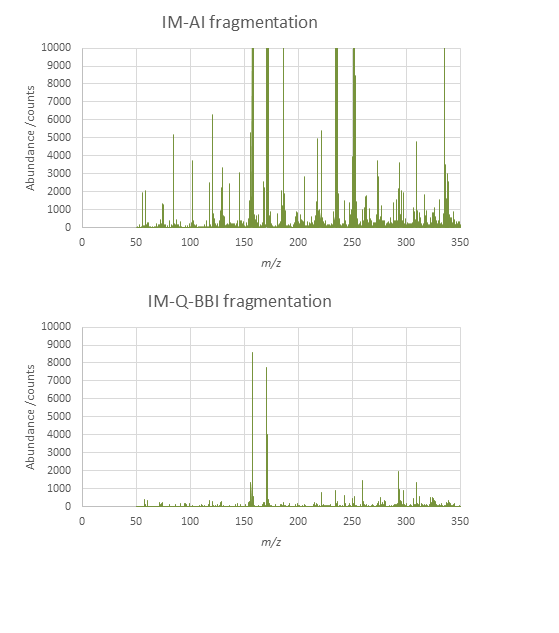

Supplement: Supplementary file 4 — Figure S3: CID fragment spectra obtained by LC‐IM‐QTOFMS in a non‐targeted IM‐AI or the IM‐Q‐BBI approach applying the fragmentation conditions described in the experimental section. The spectra where extracted in the retention time interval of 1.15–1.45 min (retention time window of glycine) of an ethanolic extract of the yeast Pichia pastoris, which was spiked with 10 μM glycine. [file RCM-33-66-s004.tif]
